# Supplementary figures and images for: New estimates of the costs of adverse events in patients with cancer
Source: PLoS One. 2025 Sep 29;20(9):e0332703. doi: 10.1371/journal.pone.0332703 (PMC12478943; doi:10.1371/journal.pone.0332703)

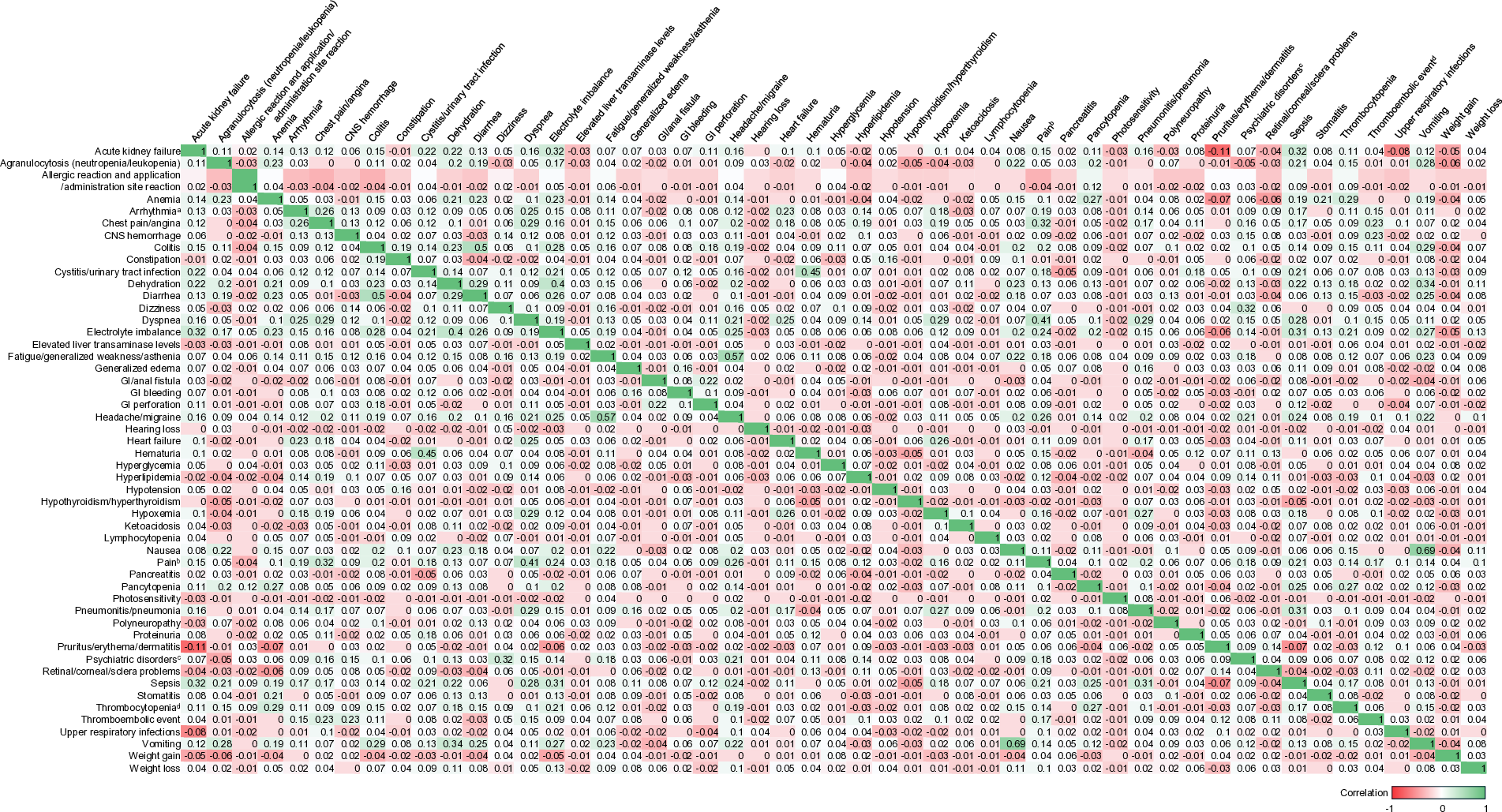

Supplement: S1 Fig — AE, adverse event; CNS, central nervous system; GI, gastrointestinal. aArrhythmia, i.e., arrhythmia/tachycardia/atrial fibrillation. bPain includes myalgia, back/musculoskeletal/laryngeal pain, pain in extremities, proctalgia, pain in limb, abdominal pain, and neuritic pain. cPsychiatric disorders include depression, anxiety, confusion, agitation, euphoria, disorientation, emotional ability, hallucinations, mood alteration, nervousness, agitation, and irritability. dThromboembolic events include pulmonary embolism, thrombotic/embolic stroke, and venous/arterial thromboembolism/embolism. (TIF) [file pone.0332703.s001.tif]

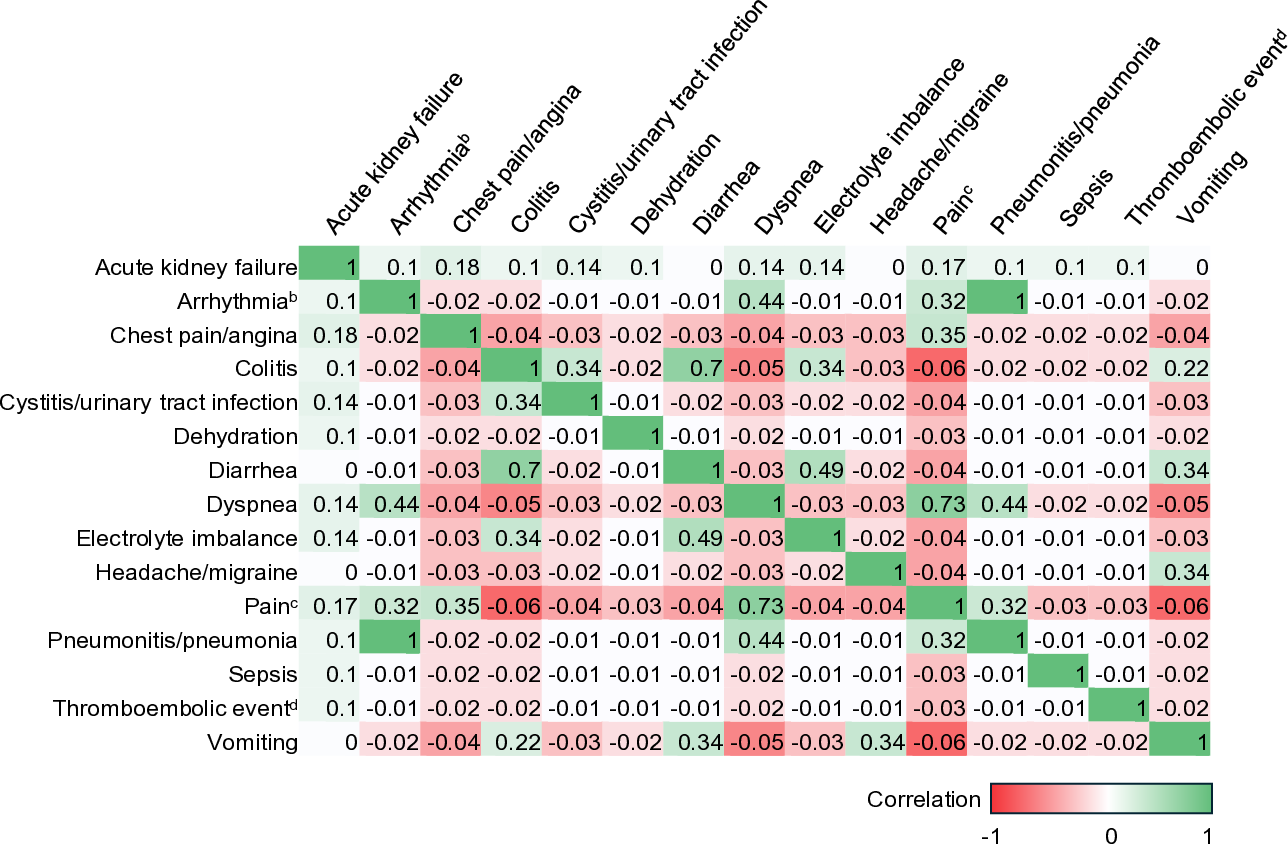

Supplement: S2 Fig — AE, adverse event. aOnly severe AEs (AEs grade 3–4) with available data were evaluated. bArrhythmia, i.e., arrhythmia/tachycardia/atrial fibrillation. cPain includes myalgia, back/musculoskeletal/laryngeal pain, pain in extremities, proctalgia, pain in limb, abdominal pain, and neuritic pain. dThromboembolic events include pulmonary embolism, thrombotic/embolic stroke, and venous/arterial thromboembolism/embolism. (TIF) [file pone.0332703.s002.tif]
