# Supplementary material for: New estimates of the costs of adverse events in patients with cancer
Source: PLoS One. 2025 Sep 29;20(9):e0332703. doi: 10.1371/journal.pone.0332703 (PMC12478943; doi:10.1371/journal.pone.0332703)
Supplement: Attachment — Submitted filename: PONE-D-24-53463_Academic editor comments.pdf [file pone.0332703.s003.pdf]

PLOS ONE

New estimates of the costs of adverse events in patients with cancer

--Manuscript Draft--

**Manuscript Number:**	PONE-D-24-53463
**Article Type:**	Research Article
**Full Title:**	New estimates of the costs of adverse events in patients with cancer
**Short Title:**	New estimates of the costs of adverse events in patients with cancer
**Corresponding Author:**	Achal Patel<br>Genentech Inc<br>South San Francisco, CA UNITED STATES OF AMERICA
**Keywords:**	healthcare economic model for cancer treatment, adverse events, cancer treatment, incremental cost of adverse events, economic burden of adverse events
**Abstract:**	The cost of adverse events (AEs) is a critical component of healthcare economic models for cancer treatment; however, few comprehensive estimates of these costs exist. Previous studies focused on costs related to specific AEs, cancer types, or treatment regimens. This study assesses the incremental costs of AEs across 13 cancer types by applying the most current diagnosis schema (International Statistical Classification of Diseases and Related Health Problems [ICD-10]) to patients diagnosed between 2016 and 2022. Data were acquired from US insurance claims and included adult patients who were diagnosed and treated for breast, pancreatic, bladder, lung, prostate, colorectal, skin, liver, head and neck, non-Hodgkin lymphoma, follicular lymphoma, multiple myeloma, or chronic lymphocytic leukemia cancer. We selected 54 AEs and 25 severe AEs (grade 3-4) for analysis based on their frequency of occurrence, relevance based on severity, and availability of ICD-10 diagnostic codes. We matched patient treatment episodes (the period from initiation of a distinct treatment regimen until discontinuation, change of treatment, or end of data availability) for patients experiencing a specific AE to treatment episodes of similar patients not experiencing that same AE in a 1:1 ratio. We generated estimates for AEs of any severity, severe AEs, and individual tumor types. Our analyses evaluate 392,566 treatment episodes from 110,791 patients. In pooled analyses across tumor types, incremental costs for AEs of any severity ranged from $293 for dehydration to $29,329 for allergic reaction. The three most-costly AEs of any severity were allergic reaction ($29,329), gastrointestinal (GI) perforation ($17,333), and ketoacidosis ($16,943). Incremental costs for severe AEs ranged from -$488 for dizziness to $19,525 for GI bleeding. The three most costly severe AEs were GI bleeding ($19,525), pain ($16,922), and nausea ($13,996). In conclusion, this study provides a comprehensive assessment of the economic burden of AEs in cancer care.
**Order of Authors:**	Achal Patel
	Robert Schuldt, PhD
	Jesse Sussell, PhD
**Opposed Reviewers:**	Nicole Engel-Nitz<br>Optum Inc<br><br>Analysis Group affiliation co-authors on Wong et al. 2018, given that this is an update to their work (https://pubmed.ncbi.nlm.nih.gov/29652926/)
**Additional Information:**	
**Question**	**Response**
**Financial Disclosure**<br><br>Enter a financial disclosure statement that describes the sources of funding for the work included in this submission. Review	Yes

Powered by Editorial Manager® and ProduXion Manager® from Aries Systems Corporation

the submission guidelines for detailed requirements. View published research articles from *PLOS ONE* for specific examples.<br><br>This statement is required for submission and **will appear in the published article** if the submission is accepted. Please make sure it is accurate.<br><br>**Funded studies**<br>Enter a statement with the following details:<br>• Initials of the authors who received each award<br>• Grant numbers awarded to each author<br>• The full name of each funder<br>• URL of each funder website<br>• Did the sponsors or funders play any role in the study design, data collection and analysis, decision to publish, or preparation of the manuscript?<br><br>Did you receive funding for this work?	
Please add funding details.<br>as follow-up to "**Financial Disclosure**<br><br>Enter a financial disclosure statement that describes the sources of funding for the work included in this submission. Review the submission guidelines for detailed requirements. View published research articles from *PLOS ONE* for specific examples.<br><br>This statement is required for submission and **will appear in the published article** if the submission is accepted. Please make sure it is accurate.	This study was funded by Genentech, Inc.

**Funded studies**<br>Enter a statement with the following details:<br>• Initials of the authors who received each award<br>• Grant numbers awarded to each author<br>• The full name of each funder<br>• URL of each funder website<br>• Did the sponsors or funders play any role in the study design, data collection and analysis, decision to publish, or preparation of the manuscript?<br><br>Did you receive funding for this work?"	
Please select the country of your main research funder (please select carefully as in some cases this is used in fee calculation).<br>as follow-up to "**Financial Disclosure**<br><br>Enter a financial disclosure statement that describes the sources of funding for the work included in this submission. Review the submission guidelines for detailed requirements. View published research articles from *PLOS ONE* for specific examples.<br><br>This statement is required for submission and **will appear in the published article** if the submission is accepted. Please make sure it is accurate.<br><br>**Funded studies**<br>Enter a statement with the following details:<br>• Initials of the authors who received each award<br>• Grant numbers awarded to each author<br>• The full name of each funder<br>• URL of each funder website<br>• Did the sponsors or funders play any role in the study design, data collection and analysis, decision to publish, or preparation of the manuscript?<br><br>Did you receive funding for this work?"	UNITED STATES - US
**Competing Interests**	I have read the journal's policy and the authors of this manuscript have the following

Powered by Editorial Manager® and ProduXion Manager® from Aries Systems Corporation

Use the instructions below to enter a competing interest statement for this submission. On behalf of all authors, disclose any competing interests that could be perceived to bias this work—acknowledging all financial support and any other relevant financial or non-financial competing interests.<br><br>This statement is required for submission and **will appear in the published article** if the submission is accepted. Please make sure it is accurate and that any funding sources listed in your Funding Information later in the submission form are also declared in your Financial Disclosure statement.<br><br>View published research articles from *PLOS ONE* for specific examples.<br><br>**NO authors have competing interests**<br><br>Enter: *The authors have declared that no competing interests exist.*<br><br>**Authors with competing interests**<br><br>Enter competing interest details beginning with this statement:<br><br>*I have read the journal's policy and the authors of this manuscript have the following competing interests: [insert competing interests here]*<br><br>* typeset	competing interests: Stock or stock options- Genentech; All support for the present manuscript (e.g., funding, provision of study materials, medical writing, article processing charges, etc.) - Genentech; current employer- Genentech
**Ethics Statement**<br><br>Enter an ethics statement for this submission. This statement is required if the study involved:<br><br>• Human participants<br>• Human specimens or tissue<br>• Vertebrate animals or cephalopods<br>• Vertebrate embryos or tissues	Data were de-identified according to Health Insurance Portability and Accountability Act patient confidentiality requirements, and institutional review board approval was not required.

• Field research<br><br>Write "N/A" if the submission does not require an ethics statement.<br><br>General guidance is provided below. Consult the submission guidelines for detailed instructions. **Make sure that all information entered here is included in the Methods section of the manuscript.**	

Powered by Editorial Manager® and ProduXion Manager® from Aries Systems Corporation

**Format for specific study types**<br><br>**Human Subject Research (involving human participants and/or tissue)**<br>• Give the name of the institutional review board or ethics committee that approved the study<br>• Include the approval number and/or a statement indicating approval of this research<br>• Indicate the form of consent obtained (written/oral) or the reason that consent was not obtained (e.g. the data were analyzed anonymously)<br><br>**Animal Research (involving vertebrate animals, embryos or tissues)**<br>• Provide the name of the Institutional Animal Care and Use Committee (IACUC) or other relevant ethics board that reviewed the study protocol, and indicate whether they approved this research or granted a formal waiver of ethical approval<br>• Include an approval number if one was obtained<br>• If the study involved *non-human primates*, add *additional details* about animal welfare and steps taken to ameliorate suffering<br>• If anesthesia, euthanasia, or any kind of animal sacrifice is part of the study, include briefly which substances and/or methods were applied<br><br>**Field Research**<br><br>Include the following details if this study involves the collection of plant, animal, or other materials from a natural setting:<br>• Field permit number<br>• Name of the institution or relevant body that granted permission	
**Data Availability**<br><br>Authors are required to make all data underlying the findings described fully available, without restriction, and from the time of publication. PLOS allows rare exceptions to address legal and ethical concerns. See the PLOS Data Policy and FAQ for detailed information.	No - some restrictions will apply

A Data Availability Statement describing where the data can be found is required at submission. Your answers to this question constitute the Data Availability Statement and **will be published in the article**, if accepted.<br><br>**Important:** Stating 'data available on request from the author' is not sufficient. If your data are only available upon request, select 'No' for the first question and explain your exceptional situation in the text box.<br><br>Do the authors confirm that all data underlying the findings described in their manuscript are fully available without restriction?	
**Describe where the data may be found in full sentences. If you are copying our sample text, replace any instances of XXX with the appropriate details.**<br><br>• If the data are **held or will be held in a public repository**, include URLs, accession numbers or DOIs. If this information will only be available after acceptance, indicate this by ticking the box below. For example: *All XXX files are available from the XXX database (accession number(s) XXX, XXX.)*.<br>• If the data are all contained **within the manuscript and/or Supporting Information files**, enter the following: *All relevant data are within the manuscript and its Supporting Information files.*<br>• If neither of these applies but you are able to provide **details of access elsewhere**, with or without limitations, please do so. For example:<br><br>*Data cannot be shared publicly because of [XXX]. Data are available from the XXX Institutional Data Access / Ethics Committee (contact via XXX) for researchers who meet the criteria for access to confidential data.*<br><br>*The data underlying the results presented in the study are available from (include the name of the third party*	The study used administrative claims data to assess the cost of adverse events across different cancers. The claims database (PharmetricsPlus) is proprietary, provided by a third-party vendor (IQVIA), and the authors do not have permission to disseminate the data without vendor approval. The study sponsor purchased access to the PharmetricsPlus data, and authors were granted access to the data on a per-project basis. Any researchers interested in obtaining the data used in the study can access the PharmetricsPlus database directly through IQVIA, under a license agreement, including the payment of appropriate license fees. Researchers can contact IQVIA via their website (https://www.iqvia.com/locations/united-states/library/fact-sheets/iqvia-pharmetrics-plus). The authors did not have any special access privileges that others would not have.

and contact information or URL).<br>• This text is appropriate if the data are owned by a third party and authors do not have permission to share the data.<br><br>* typeset	
Additional data availability information:	

Powered by Editorial Manager® and ProduXion Manager® from Aries Systems Corporation

Emily Chenette, PhD
Editor-in-Chief
PLOS One

Dear Dr Chenette,

On behalf of my co-authors, I am pleased to submit our manuscript entitled, "**New estimates of the costs of adverse events in patients with cancer**" for consideration as an original article in *PLOS ONE*.

In cancer treatment, costs related to adverse events (AEs) are an important consideration and a critical component for robust healthcare economic modeling. Previous studies have focused on costs related to specific cancer types, AEs or treatment regimens, but failed to provide comprehensive estimates of cancer treatment costs. In this study, we improve and expand upon the design of Wong et al. (Wong et al., *PLOS ONE*, 2017) by applying a more-recent patient data set (patients diagnosed 2016-2022 vs 2006-2015) and a more modern diagnosis schema (International Statistical Classification of Diseases and Related Health Problems [ICD-10] vs ICD-9) to generate estimates for more AEs (54 vs 36), which are also stratified across a larger number of distinct cancer types (13 vs 8).

From 110,791 patients, we evaluated a total of 392,566 treatment episodes. We selected 54 AEs and 25 severe AEs (grade 3-4) for analysis. We generated estimates for AEs of any severity, severe AE, and individual tumor types. In pooled analyses across tumor types, the most costly AEs of any severity per treatment episode were allergic reaction ($29,329 [USD]), gastrointestinal (GI) perforation ($17,333) and ketoacidosis ($16,943). The most costly severe AEs per treatment episode were GI bleeding ($19,525), pain ($16,922) and nausea ($13,996). The average cost of an AE of any severity versus severe AE was $6,835 versus $9,518. Additionally, we evaluated the correlation of co-occurrence between AEs to offer a more robust estimate of incremental costs of any given AE compared with the previous Wong et al. methodology.

We believe this manuscript provides a timely and important advance in the methodology for cost estimations of AEs in cancer treatment. The results of this study will serve as a resource for the research community engaged in the development of more comprehensive, oncology-focused health economic models.

The findings of this study have not been published previously, nor are they under consideration for publication elsewhere. All authors have reviewed and approved the manuscript. We thank you in advance for your consideration and look forward to your response.

Sincerely,

Achal Patel, PhD
Genentech Inc, 1 DNA Way
South San Francisco, CA 94080 USA
Patel.achal@gene.com

Manuscript

Click here to access/download;Manuscript;PLos_CostofAE_Patel_MS_FINAL

New estimates of the costs of adverse events in patients with cancer

(68/250 characters)

Achal Patel, PhD,[1]* Robert Schuldt, PhD,[1] Jesse Sussell, PhD[1]

[1] Department of Medical Affairs, Genentech, South San Francisco, CA, USA

*Corresponding author:

E-mail: patel.achal@gene.com

Short title: New estimates of the costs of adverse events in patients with cancer (68/100 characters)

Target journal: PLOS One

Article type: Original investigation

Abstract word count: 298/300

Figure and table counts: 4 figures, 4 tables

References: 12

Supplementary materials: 2

Abstract (298/300 words)

The cost of adverse events (AEs) is a critical component of healthcare economic models for cancer treatment; however, few comprehensive estimates of these costs exist. Previous studies focused on costs related to specific AEs, cancer types, or treatment regimens. This study assesses the incremental costs of AEs across 13 cancer types by applying the most current diagnosis schema (International Statistical Classification of Diseases and Related Health Problems [ICD-10]) to patients diagnosed between 2016 and 2022. Data were acquired from US insurance claims and included adult patients who were diagnosed and treated for breast, pancreatic, bladder, lung, prostate, colorectal, skin, liver, head and neck, non-Hodgkin lymphoma, follicular lymphoma, multiple myeloma, or chronic lymphocytic leukemia cancer. We selected 54 AEs and 25 severe AEs (grade 3-4) for analysis based on their frequency of occurrence, relevance based on severity, and availability of ICD-10 diagnostic codes. We matched patient treatment episodes (the period from initiation of a distinct treatment regimen until discontinuation, change of treatment, or end of data availability) for patients experiencing a specific AE to treatment episodes of similar patients not experiencing that same AE in a 1:1 ratio. We generated estimates for AEs of any severity, severe AEs, and individual tumor types. Our analyses evaluate 392,566 treatment episodes from 110,791 patients. In pooled analyses across tumor types, incremental costs for AEs of any severity ranged from $293 for dehydration to $29,329 for allergic reaction. The three most-costly AEs of any severity were allergic reaction ($29,329), gastrointestinal (GI) perforation ($17,333), and ketoacidosis ($16,943). Incremental costs for severe AEs ranged from -$488 for dizziness to $19,525 for GI bleeding. The three most costly severe AEs were GI bleeding ($19,525), pain ($16,922), and nausea ($13,996). In conclusion, this study provides a comprehensive assessment of the economic burden of AEs in cancer care.

1. Introduction

To improve our understanding of healthcare and health policy, economic models provide insight on the tradeoff between costs and benefits of different therapeutic interventions. In many countries, sophisticated health economic models are required for regulatory approval, along with a demonstration of clinical efficacy. In general, this takes the form of a cost-effectiveness or cost-utility analysis, in which the estimated costs (e.g. cost of additional health screening or preventative medications) and benefits (e.g. overall survival or quality-adjusted life years) of a novel therapeutic approach are weighed against each other. These costs and benefits are usually estimated relative to some comparator, typically the current standard-of-care therapy. For example, in oncology, the costs and benefits of targeted therapy have frequently been modeled relative to chemotherapy or older generations of targeted therapy. In cost-benefit analyses, it is important to measure all costs of a therapeutic approach in addition to the cost of medications [1]. Understanding the tradeoffs between the costs and benefits of different therapies helps payers make decisions under scarcity, thus maximizing the health benefit obtained by the population from a finite payer budget.

In oncology, the primary driver of cost differences is the relative price of the therapeutic regimens. However, adverse events (AEs) represent an important ancillary source of cost differences. For example, an economic study on metastatic melanoma found that the incremental costs associated with specific treatment-related AEs (TRAEs) were high, especially so for AEs related to metabolic and nutritional disorders ($9,135 per 30-day period) [2]. Another direct cost study, related to systemic therapies for melanoma, highlighted large cost differences between different types of grade 3 or 4 TRAEs, especially those that necessitated hospitalizations or expensive outpatient care [3]. Additionally, a cost analysis of immunotherapies in renal cell carcinoma demonstrated a wide spectrum of all-cause and TRAE costs differences between regimens [4].

Conceptually, the expected value cost of an AE is the product of the event rate over time and the average payer cost of treating the event when it occurs. However, there is frequently a paucity of information related to the estimated average cost of treating any individual AE. Parameterization of an economic model with accurate AE cost data can be difficult for a number of reasons: studies that evaluate the costs of AEs frequently only consider AEs experienced by patients with a single tumor type (reducing external validity) and are often conducted using a variety of methodologies (limiting comparability) or use a micro-costing approach, which is known to underestimate total associated costs [5-7]. Frequently, direct or modeled estimates of the incremental cost of treating specific AEs, such as dizziness or sepsis, are not available. When this occurs, the research community frequently assumes equivalence between the cost of treating plausibly similar AEs (e.g. leukopenia and neutropenia), potentially introducing bias into study results [8-10]. In 2018, Wong et al. significantly improved the data available to the modeling community by publishing a set of estimates of AE costs [5]. Estimates from this study were generated through analysis of insurance claims data and included estimates for a pooled sample of patients with cancer, both for patients with overall AEs and for patients with severe (grade 3-4) AEs. That study stratified results for eight different tumor types.

In the present study, we replicate the broad design of Wong et al. with an application to more-recent patient data (patients diagnosed 2016-2022 vs 2006-2015) [5]. We also applied a more modern diagnosis schema (International Statistical Classification of Diseases and Related Health Problems [ICD] -10 vs ICD-9) to generate estimates for more AEs (54 vs 36), which are also stratified across a larger number of distinct tumor types (13 vs 8). The results of this study are intended to serve as a resource for the research community engaged in the development of oncology-focused health economic models.

2. Materials and methods

2.1 Data source

This study was conducted using the IQVIA PharMetrics® Plus dataset, which includes medical and pharmacy claims for largely commercially insured patients and their dependents, as well as patients with Managed Medicare and Medicare Advantage insurance in the United States.

2.2 Overview of study design

In this study, we sought to estimate the incremental cost impact of experiencing different AEs on the total cost of care as measured in healthcare claims data. We considered three different levels of analysis. Our primary analysis was to estimate the cost of individual AEs (e.g. nausea) on healthcare costs in patients with cancer, pooled across different tumor types. In a secondary analysis and consistent with Wong et al. [5], we considered the specific costs of severe AEs (grades 3-4) in the same pooled sample of patients with cancer. In a tertiary analysis, we considered the costs of AEs in 13 groups, stratified by tumor type: breast (ICD-10-CM C50.91), pancreatic (ICD-10-CM C25.1), bladder (ICD-10-CM C67.9), lung (ICD-10-CM C34.9), prostate (ICD-10-CM C61.0), colorectal (ICD-10-CM C18.9), skin (ICD-10-CM C44.9), liver (ICD-10-CM C22.0), head and neck (ICD-10-CM C76.0), non-Hodgkin lymphoma (ICD-10-CM C85.9), follicular lymphoma (ICD-10-CM C82.9), multiple myeloma (ICD-10-CM C90.0), and chronic lymphocytic leukemia (ICD-10-CM C91.1). In each of these analyses, we estimated the incremental cost impact of the AE in question by comparing the total healthcare costs incurred by a “treatment group patient” (i.e. a patient experiencing a given AE type) with those of a “matched control group patient” within a given treatment episode. More details on the matching procedures and treatment episode definitions are described below. To reduce potential bias, we excluded all costs associated with antineoplastic treatment, including surgery, radiotherapy, and pharmaceutical costs for oncologic therapies.

2.3 Patient selection

2.3.1 Treatment group selection

Data were de-identified according to Health Insurance Portability and Accountability Act patient confidentiality requirements, and institutional review board approval was not required. Adult patients were selected for inclusion in the treatment group if they were initially diagnosed with one of the 13 cancer types included in this study between 2016 and 2022 and if they were subsequently diagnosed with one of the 54 AEs of interest within a given treatment episode (see below for treatment episode definitions) (**Fig 1**).

Fig 1. Attrition table. [a]The 110,791 patients contributed 392,566 treatment episodes.

2.3.2 Control group selection

The inclusion criteria for the matching control group were the same as the treatment group, except patients in the control group were required to *not* have a within-treatment episode diagnosis for the AE of interest. For example, for a patient in the treatment group who had the AE *nausea,* the pool of potential matched controls was required to not have nausea but could have any of the other AEs considered in this study.

2.4 Outcomes

The study outcome is the average incremental difference in total medical spending between the matched pairs of patients, each of which is defined by either experiencing (treatment group) or not experiencing (matched controls group) the AE of interest during the treatment episode being considered.

2.5 Treatment episode definitions

Following Wong et al., each patient was allowed to complete multiple treatment episodes for the analysis [5]. The start of a treatment episode was defined as the initiation of a distinct antineoplastic regimen. The end of a treatment episode was marked by the occurrence of any of the following: discontinuation of the treatment episode (i.e. gap of ≥45 days in the use of all agents that were part of the treatment regimen), initiation of a new antineoplastic regimen (resulting in the start of a new treatment episode), administrative censoring of the patient (end of continuous enrollment), or the end of data availability.

2.6 Classification and matching of treatment episodes

These methods were described in detail in Wong et al. and are briefly summarized here [5]. Treatment episodes were classified into two groups: treatment episode with AE and treatment episode without AE. Treatment episode with AE was defined as a diagnosis of an AE recorded during the treatment episode. Treatment episode without AE was defined as the absence of a diagnostic code for the particular AE of interest during the duration of the treatment episode or the 12-month period preceding the start date of the treatment episode.

The set of AEs included in this study was selected first by examining clinical practice guidelines for the types of treatments used for the cancers included in this study. From the list of 361 AEs mentioned in the product inserts of these treatments, AEs were selected/combined for this study based on the frequency of mention, their relevance based on severity, and the availability of respective diagnostic codes from ICD-10-CM. A total of 54 AEs associated with these treatments were included in this study. Following the literature [5,11,12], severe AEs were defined as AEs that required an inpatient stay as recorded by a health insurance claim. AEs of any severity were defined as AEs coded with an ICD-10-CM diagnosis and recorded on a health insurance claim.

Treatment episodes for an AE were matched to similar treatment episodes without that same AE in a 1:1 ratio. A risk to the validity of comparing healthcare costs within treatment episodes is that the duration of treatment episodes will vary from patient to patient; as a result, the time at risk for cost accrual will differ between patients. To control for this potential bias, the duration of the treatment episodes for patients in the matched control group was truncated to be equal to the duration of the treatment episode for patients experiencing the AE of interest (**Fig 2**). When the control group patient's treatment episode duration was shorter (as opposed to longer) than the treatment group patient's treatment episode duration, that control group member was discarded and a new control was selected through matching. Treatment episodes were matched separately for each AE. Treatment episodes could be matched only once for a specific AE, but they could be matched more than once across AEs.

Fig 2. Adverse event (AE) occurrence in the context of distinct treatment episodes[5].

[a]Treatment episode start date was defined as the date of the initiation of the first antineoplastic agent as part of a treatment regimen. [b]Treatment episode end date was defined as the first of the following events: discontinuation of the treatment regimen (i.e. a gap of ≥45 days in the use of all agents in the treatment), change in treatment regimen, the end of health plan enrollment, or the end of data availability.

2.7 Statistical considerations

In the main analysis (assessment of AE costs in a pooled population of patients with cancer), three complementary bias reduction techniques were used. First, patients were exact matched by tumor type. For a patient with tumor type *i,* it was required that their matched control also be diagnosed with tumor type *i.* Patients with multiple cancer diagnoses (e.g. breast and lung) were permitted but matching used the primary diagnosis. Second, for any patient in the treatment

group, selection of the paired patient in the matched control group occurred via propensity score matching. Nearest-neighbor matching was used to match treatment and control in a 1:1 ratio with a caliper of 0.2. The following factors were used to determine the propensity score: age, sex, US region, payer type (insurance), product type (insurance), number of patient episodes, line of therapy, index year line of therapy, regimen line of therapy number, metastatic diagnosis pre-Index, adjuvant status pre-index (surgical), stem cell transplant during treatment episode (indicator), and radiotherapy during treatment episode (indicator). Third, following matching, the incremental difference in healthcare costs was estimated in an outcome model that controlled for the count of co-occurring AEs in both members of the matched pair. For example, in estimating the incremental cost impact of nausea, the outcome model incorporated as a covariate the co-occurrence of the 47 other AEs within the treatment episode. This covariate was incorporated using a flexible cubic spline approach. This aspect was included in the design because co-occurrence of AEs is plausibly correlated—in some, cases strongly so. For example, patients with breast cancer who experienced nausea were more likely to experience vomiting and vice versa (**S1 Fig**). The novel use of flexible cubic splines to model the correlation structure across AEs offers a more robust estimate of the incremental cost of any given AE than with the approach by Wong et al.

For the secondary analysis (cost estimates for patients experiencing severe AEs), two covariate factors were included: the count of co-occurring severe AEs and the count of co-occurring AEs, regardless of severity. For example, severe dyspnea and severe pain were correlated in patients with breast cancer (**S2 Fig**). For the tertiary analysis (within-tumor stratified cost estimation), exact matching based on tumor type is implied by design. To perform the propensity score match in the AE analysis, a minimum N value of 100 treatment episodes was required. For the subsequent outcomes model, only AEs with >50 matched pairs (100 treatment episodes) were reported.

3. Results

3.1 Characteristics across matched treatment episodes

The selected 110,791 patients had a total of 392,566 treatment episodes across 54 AEs (**Table 1**) during the study period from October 1, 2015, to June 30, 2022. The median age was 60 years and 58.3% were females. The most frequent primary cancer was breast cancer (29.9%), followed by cancer of the lung (14.2%), colorectal (11.5%), skin (10.9%), non-Hodgkin lymphoma (6.8%), head and neck (5.3%), pancreatic (5.1%), prostate (4.7%), liver (3.8%), multiple myeloma (3.6%), bladder (2.6%), chronic lymphocytic leukemia (0.9%), and follicular lymphoma (0.7%).

Table 1: Treatment episodes characteristics

**Treatment episodes[a]**	392,566
**Age, median (IQR)**	60 (53, 64)
**Female, n (%)**	228,790 (58.3)
**US Region, n (%)**	
East	69,160 (17.6)
Midwest	108,657 (27.7)
South	159,097 (40.5)
West	55,641 (14.2)
Other	11 (<0.1)
**Product type, n (%)**	
Consumer directed	9,099 (2.3)
HMO	72,162 (18.4)
POS	23,463 (6.0)
PPO	281,922 (71.8)
Unknown	5,920 (1.5)
**Payer type, n (%)**	
Commercial	225,149 (57.4)
Medicare Advantage	36,525 (9.3)
Medicare Supplemental	17,086 (4.4)

Self-pay	111,409 (28.4)
Unknown	2,397 (0.6)
**Treatment type, n (%)**	
Metastatic pre-treatment	220,748 (56.2)
Adjuvant therapy pre-treatment	114,051 (29.1)
**No. of episodes, n (%)**	
1	172,176 (43.9)
2	115,546 (29.4)
3	104,844 (26.7)
**LOT index year, n (%)**	
2016	6,955 (1.8)
2017	72,665 (18.5)
2018	82,780 (21.1)
2019	88,387 (22.5)
2020	83,708 (21.3)
2021	57,587 (14.7)
2022	484 (0.1)
**LOT number, n (%)**	
1	287,210 (73.2)
2	78,246 (19.9)
3	27,110 (6.9)
**Type of studied cancer, n (%)**	
Breast	117,322 (29.9)
Lung	55,834 (14.2)
Colorectal	45,051 (11.5)
Skin	42,804 (10.9)
Non-Hodgkin lymphoma	26,610 (6.8)
Head and neck	20,947 (5.3)
Pancreatic	20,149 (5.1)
Prostate	18,357 (4.7)
Liver	15,070 (3.8)
Multiple myeloma	13,976 (3.6)
Bladder	10,058 (2.6)
CLL	3,518 (0.9)
Follicular lymphoma	2,870 (0.7)

CLL, chronic lymphocytic leukemia; HMO, health maintenance program; LOT, line of therapy; POS, point of service; PPO, preferred provider organization.

[a]All percentages were calculated with the number of treatment episodes in the denominator. Treatment episodes occurred in 110,791 patients.

The number of treatment episodes matched across AEs ranged from 241,487 (pain) to 135 (encephalopathy) (**Table 2**). The five most common AEs of any severity were pain, headache/migraine, vomiting, psychiatric disorders, and colitis (**Table 2**).

Table 2: Prevalence and characteristics of matched treatment episodes by AE.

Adverse event	Proportion of episodes with AE of any severity (among all episodes), %	Matched episodes, n
Acute kidney failure	16.1	63,387
Agranulocytosis (neutropenia/leukopenia)	18.3	71,704
Allergic reaction and application/administration site reaction	0.4	1,392
Anemia	12.3	48,432
Arrhythmia	14.9	58,548
Chest pain/angina	26.4	103,722
CNS hemorrhage	4.9	19,283
Colitis	28.8	113,022
Constipation	3.6	14,125
Cystitis/urinary tract infection	12.4	48,804
Dehydration	23.0	90,246
Diarrhea	4.9	19,367
Dizziness	7.6	29,794
Dyspnea	23.2	91,194
Electrolyte imbalance	27.0	105,870
Elevated liver transaminase levels	0.8	3,307
Encephalopathy	<0.1	135
Extravasation	<0.1	187
Fatigue/generalized weakness/asthenia	28.7	112,659
Generalized edema	1.0	3,771
GI/anal fistula	0.4	1,534
GI bleeding	2.3	8,970
GI perforation	0.6	2,496
Headache/migraine	53.8	211,192
Hearing loss	0.1	546
Heart failure	1.7	6,486
Hematuria	13.8	54,076
Hyperglycemia	3.8	15,072
Hyperlipidemia	27.6	108,210
Hypertension	0.2	758

Hypotension	0.3	1,330
Hypothyroidism/hyperthyroidism	1.3	5,252
Hypoxemia	4.7	18,466
Ketoacidosis	0.3	1,222
Lymphocytopenia	0.5	1,942
Nausea	22.3	87,653
Pain	61.5	241,487
Pancreatitis	0.9	3,457
Pancytopenia	7.8	30,551
Photosensitivity	0.1	508
Pneumonitis/pneumonia	10.8	42,277
Polyneuropathy	6.6	25,990
Proteinuria	5.2	20,291
Pruritus/erythema/dermatitis	13.8	54,164
Psychiatric disorders	36.1	141,628
Retinal/corneal/sclera problems	5.8	22,931
Sepsis	11.7	45,821
Stomatitis	2.8	10,849
Thrombocytopenia	13.1	51,453
Thromboembolic event	23.5	92,270
Upper respiratory infections	9.0	35,289
Vomiting	36.3	142,541
Weight gain	0.8	2,961
Weight loss	7.4	28,873

AE, adverse event; GI, gastrointestinal; CNS, central nervous system.

~~3.[illegible] Healthcare costs~~

The incremental healthcare costs related to AEs of any severity ranged from $293 for dehydration to $29,329 for allergic reaction and application/administration site reaction (**Fig 3**). The top five most costly AEs of any severity were allergic reaction and application/administration site reaction ($29,329), gastrointestinal (GI) perforation ($17,333), ketoacidosis ($16,943), lymphocytopenia ($14,845), and GI bleeding ($13,427) (**Fig 3**).

Fig 3: Incremental costs per matched treatment episode[a]—AEs of any severity, within the pooled sample of patients with cancer. Incremental costs reported as medians (95%

confidence interval) in US dollars. AE, adverse event; GI, gastrointestinal; CNS, central nervous system. [a]Only AEs with >50 matched treatment episodes were included.

The incremental healthcare costs associated with severe AEs ranged from -$488 for dizziness to $19,525 for GI bleeding (**Fig 4**). There was no estimate for severe allergic reaction and severe application/administration site reaction to compare with AEs of any severity. The five most costly severe AEs were GI bleeding ($19,525), pain ($16,922), nausea ($13,996), chest pain/angina ($13,071), and headache/migraine ($11,558) (**Fig 4**).

Fig 4: Incremental costs per matched treatment episode[a]—severe AEs, within the pooled sample of patients with cancer. Incremental costs reported as medians (95% confidence interval) in US dollars. AEs, adverse events; CNS, central nervous system; GI, gastrointestinal. [a]Only severe AEs (grade 3-4) with >50 matched treatment episodes were included.

For common cancer types that represented greater than or equal to 6% of the selected patient population (breast [29.9%], lung [14.2%], colorectal [11.5%], skin [10.9%], non-Hodgkin lymphoma [6.8%]), GI perforation and generalized edema were commonly the most costly AEs of any severity (**Table 3**). Among these common cancer types, dehydration and diarrhea were generally the least costly AEs of any severity (**Table 3**). For rarer cancer types (less than 6% of selected patient population) including head and neck (5.3%), pancreatic (5.1%), prostate (4.7%), liver (3.8%), multiple myeloma (3.6%), bladder (2.6%), CLL (0.9%) and follicular lymphoma (0.7%), cost estimates for AEs of any severity had greater variance and were difficult to classify. (**Table 4**). For example, headaches/migraines cost more than $20,000 in follicular lymphoma, multiple myeloma, and CLL, but cost less than $1,400 in head and neck and breast

cancers (**Table 4**). This is likely a result of the relatively smaller number of matched pairs for these tumor types, and the resulting wider confidence intervals for estimates of mean cost differences.

Table 3: Incremental costs (in US dollars) per treatment episode—AEs of any severity, stratified by common tumor type (≥6% of selected patients). Each n indicates the number of treatment episodes.

**Adverse event, $**	**Breast (n=117,322)**	**Lung (n=55,834)**	**Colorectal (n=45,051)**	**Skin (n=42,804)**	**Non-Hodgkin lymphoma (n=26,610)**
Acute kidney failure	9,475.80	4,296.71	7,021.93	3,620.30	11,653.96
Agranulocytosis (neutropenia/leukopenia)	5,411.14	1,958.32	10,655.26	3,952.56	9,988.52
Allergic reaction and application/administration site reaction	13,824.52	-	-	47,129.81	27,428.70
Anemia	5,688.15	3,938.98	2,533.94	6,611.12	7,318.33
Arrhythmia	9,038.30	11,545.09	5,452.32	7,889.00	11,430.56
Chest pain/angina	10,935.38	6,124.61	-1,816.48	5,971.03	7,536.21
CNS hemorrhage	11,399.39	6,857.51	4,605.40	7,457.49	17,481.04

Colitis	7,709.95	4,298.86	-1,681.88	7,782.64	12,672.76
Constipation	10,385.35	7,560.57	6,525.89	5,628.82	3,210.76
Cystitis/urinary tract infection	6,131.37	4,835.76	-1,071.79	6,047.18	10,568.27
Dehydration	1,579.51	1,630.03	-2,129.97	345.83	3,602.93
Diarrhea	-1,558.50	1,380.92	-1,373.45	8,594.31	1,905.12
Dizziness	7,924.91	5,243.67	60.19	5,713.68	9,491.90
Dyspnea	7,171.88	3,517.05	853.83	4,181.86	5,654.68
Electrolyte imbalance	7,419.89	4,876.74	3,726.50	5,937.37	9,923.48
Elevated liver transaminase levels	6,399.11	4,251.40	477.7	11,832.41	21,070.65
Encephalopathy	3,640.17	-	-	-	-
Extravasation	-1,434.11	-	-	-	-
Fatigue/generalized weakness/asthenia	7,087.50	4,256.11	-195.05	5,740.71	6,951.45
Generalized edema	10,854.62	11,208.50	22,591.37	15,022.26	11,601.46
GI/anal fistula	5,411.24	-	12,618.26	-445.38	-
GI bleeding	14,593.36	8,837.05	3,026.45	1,569.61	12,653.28
GI perforation	19,284.84	24,404.59	16,826.43	12,555.16	-

Headache/migraine	7,298.08	4,588.67	-1,248.90	6,611.51	4,544.70
Hearing loss	-221.24	-	-	-	-
Heart failure	974.21	5,963.99	-1,960.04	8,333.34	10,971.40
Hematuria	8,171.99	5,595.13	1,164.81	4,605.42	8,525.97
Hyperglycemia	9,776.95	8,137.74	9,353.71	10,280.18	18,360.55
Hyperlipidemia	5,524.12	9,308.11	4,308.34	2,690.72	5,619.93
Hypertension	5,197.66	-	-	-	-
Hypotension	8,955.82	10,838.78	-	-	-
Hypothyroidism/hyperthyroidism	10,652.80	5,312.44	3,709.01	10,124.72	8,394.91
Hypoxemia	11,200.79	7,093.09	1,082.07	10,501.76	5,204.08
Ketoacidosis	11,745.78	6,912.14	-	24,023.80	25,087.33
Lymphocytopenia	9,500.85	16,693.68	-	7,294.63	16,588.59
Nausea	5,861.58	-94.09	1,006.13	3,731.74	4,718.90
Pain	5,791.95	8,259.50	2,427.53	3,825.56	4,852.23
Pancreatitis	13,151.31	7,844.07	3,609.34	12,546.36	23,236.33
Pancytopenia	6,537.11	2,986.94	4,534.17	1,271.99	14,102.22
Photosensitivity	4,785.99	-	-	-6,454.32	-
Pneumonitis/pneumonia	7,964.50	5,779.83	8,017.54	5,939.67	10,085.17

Polyneuropathy	3,069.89	6,076.00	-522.9	5,377.44	11,457.29
Proteinuria	7,432.77	4,307.68	3,506.38	8,062.66	15,144.84
Pruritus/erythema/dermatitis	9,342.04	3,082.76	3,767.29	3,223.33	10,503.02
Psychiatric disorders	9,875.05	7,780.56	2,686.29	6,394.39	9,015.84
Retinal/corneal/sclera problems	8,106.87	7,427.44	-4,373.84	8,513.86	2,630.14
Sepsis	10,800.03	7,733.94	6,745.63	9,806.11	9,161.80
Stomatitis	10,288.75	18,954.48	-2,747.67	-1,039.15	18,552.18
Thrombocytopenia	5,457.08	5,851.23	3,806.49	7,461.67	11,633.31
Thromboembolic event	11,936.81	6,992.02	5,022.27	7,183.91	11,945.86
Upper respiratory infections	10,994.18	8,342.63	4,775.90	6,117.57	11,339.64
Vomiting	5,057.79	2,382.06	556.34	5,097.29	6,711.70
Weight gain	8,702.54	9,840.76	3,493.99	9,016.90	3,701.82
Weight loss	7,743.57	5,938.27	4,642.31	6,026.81	3,670.13

AEs, adverse events; CNS, central nervous system; GI, gastrointestinal.

Legend:

	≥1,500 matched treatment episodes
	1,000-1,500 matched treatment episodes

	500-1,000 matched treatment episodes
	<500 matched treatment episodes
-	Insufficient sample size for robust statistical model

Table 4: Incremental costs (in US dollars) per treatment episode—AEs of any severity in rarer tumor types (<6% of selected patients). Each n indicates the number of episodes.

Adverse event, $	Head and neck (n=20,947)	Pancreatic (n=20,149)	Prostate (n=18,357)	Liver (n=15,070)	Multiple myeloma (n=13,976)	Bladder (n=10,058)	CLL (n=3,518)	Follicular lymphoma (n=2,870)
Acute kidney failure	804.59	6,873.79	1,214.69	4,418.82	12,172.64	5,505.64	10,542.31	10,177.87
Agranulocytosis (neutropenia/leukopenia)	2,039.82	2,240.46	4,449.51	3,954.71	12,686.58	7,568.82	7,080.15	13,552.53
Allergic reaction and application/administration site reaction	-	-	-	-	-	-	-	-
Anemia	-406.6	-42.95	6,698.70	1,841.50	14,839.62	-64.94	11,868.51	11,340.28
Arrhythmia	1,665.04	-1,610.62	219.38	8,393.67	14,940.73	-2146.6	20,831.02	10,249.23
Chest pain/angina	-76.62	-2,262.24	110.16	4,077.56	13,203.75	-1,033.65	8,670.18	3,921.35

CNS hemorrhage	-3,488.47	7,509.90	-1,547.24	-2,155.85	8,377.84	3,900.41	5,210.59	-8,181.28
Colitis	1,973.17	-1,537.30	4,992.71	3,976.60	12,154.94	1,491.64	21,877.14	18,344.75
Constipation	2,496.70	2,855.54	13,173.95	6,397.73	20,246.17	6,020.40		-
Cystitis/urinary tract infection	1,792.09	694.71	3,041.24	-5,555.58	15,137.92	1,764.07	10,310.57	4,886.91
Dehydration	-2,230.23	-6,344.91	-2,666.02	2,611.52	11,060.28	453.69	2,695.24	2,752.78
Diarrhea	1,420.99	2251.22	-4,429.23	-1,418.65	15,481.33	3,945.58	-	-
Dizziness	-3,011.95	1,866.03	-1,228.78	3,701.37	2,423.04	3,800.75	118.68	11,823.83
Dyspnea	6,295.22	1,689.75	3,321.61	-109.79	7,863.32	4,990.78	14,636.56	11,318.53
Electrolyte imbalance	3,160.36	-2,440.40	4,212.12	2,115.97	13,580.58	5,776.90	6,142.57	-48.54
Elevated liver transaminase levels	-	-9,742.78	-	2,891.66	-	-11,094.09	-	-
Encephalopathy	-	-	-	-	-	-	-	-
Extravasation	-	-	-	-	-	-	-	-

Fatigue/generalized weakness/asthenia	1,436.58	-2,347.12	891.76	-3,866.08	13,313.77	1,782.27	3,336.62	8,437.40
Generalized edema	-	10,617.03	-	-8,744.58	10,512.78	-	-	-
GI/anal fistula	-	-	-	-	-	-	-	-
GI bleeding	11,602.41	15,831.53	16,300.97	24,502.38	24,550.20	1,344.68	-	-
GI perforation			-				-	-
Headache/migraine	1,365.96	-7,468.66	-717.42	7,955.41	15,134.68	2,575.68	29,928.56	20,366.70
Hearing loss	-	-	-	-	-	-	-	-
Heart failure	-	5,761.81	5,392.52	-3,446.77	14,628.52	10,363.86	-	-
Hematuria	6,101.76	764.03	2,491.35	3,615.84	9,211.66	187.52	10,217.14	4,415.18
Hyperglycemia	2,850.92	18,988.70	-25.39	283.91	7,347.12	6,726.98	25,720.01	-
Hyperlipidemia	6,395.38	559.61	-1,173.85	9,199.43	9,194.61	-3,273.41	817.15	-3,180.27
Hypertension	-	-	-	-	-	-	-	-
Hypotension	-	-	-	-	-	-	-	-

Hypothyroidism/hyperthyroidism	7,576.61	9,884.48	-1,481.53	20,163.43	-	5,085.67	-	-
Hypoxemia	7,500.83	2,495.82	15,791.06	6,937.04	8,570.51	18,050.72	26,552.25	-
Ketoacidosis	-	-	-	-	-	-	-	-
Lymphocytopenia	-	-	-	-	16,017.20	-	-	-
Nausea	-505.87	1,096.80	-4,461.34	1,682.87	9,332.43	6,715.52	7,420.53	5,536.50
Pain	2,727.10	-22,365.09	1,362.17	3,813.43	14,820.62	-1,926.82	10,211.26	13,029.38
Pancreatitis	10,054.42	-7,174.85	-	31,255.48				
Pancytopenia	4,808.98	7,392.54	-3,629.80	-5,703.53	15,449.56	7,970.92	30,060.42	31,090.31
Photosensitivity	-	-	-	-	-	-	-	-
Pneumonitis/pneumonia	2,505.81	1,793.50	1,281.95	5,050.74	9,608.83	6,542.78	9,984.43	14,530.47
Polyneuropathy	-3,399.86	7,757.05	20,048.05	-6,199.96	9,295.65	11,393.44	-	-
Proteinuria	-409.41	2,459.15	-244.88	641.47	13,645.66	2,556.03	19,384.97	-6,936.97

Pruritus/erythema/dermatitis	-148.33	-1,819.89	3,262.99	1,524.71	15,424.32	11.75	2,844.56	-132.15
Psychiatric disorders	0.56	4,467.60	2,033.66	1,195.91	14,167.17	3,521.95	7,264.27	2,022.51
Retinal/corneal/sclera problems	5,923.40	-1,286.34	1,760.51	1,408.92	15,425.43	4,871.50	-4,395.39	-2,642.32
Sepsis	7,747.13	501.2	6,206.16	6,137.18	11,509.10	-4,370.11	11,268.17	13,707.65
Stomatitis	2,171.06	1,301.65	-	-7,743.01	-3,570.14	-	-	-
Thrombocytopenia	-436.77	3,764.00	2,336.54	6,577.56	15,257.03	4,490.10	14,993.78	8,338.45
Thromboembolic event	9,942.36	-670.08	4,448.76	2,101.70	14,165.09	3,023.13	13,132.48	7,684.95
Upper respiratory infections	131.44	-40.5	1,914.73	7,946.03	11,595.23	2,467.73	11,994.77	1,645.34
Vomiting	132.3	-347.75	3,631.26	3,634.01	11,667.49	2,558.23	7,184.36	2,251.74
Weight gain	-	-	3,338.68	-	-	-	-	-

Weight loss	1,894.17	464.51	5,838.23	-2,911.82	13,404.28	1,905.90	8,417.18	-17,747.78

AEs, adverse events; CLL, chronic lymphocytic leukemia; CNS, central nervous system; GI, gastrointestinal.

Legend:

	≥1,500 matched treatment episodes
	1,000-1,500 matched treatment episodes
	500-1,000 matched treatment episodes
	<500 matched treatment episodes
-	Insufficient sample size for robust statistical model

4. Discussion

Following guidance by the Professional Society for Health Economics and Outcomes Research, it is critical to evaluate the incremental cost differences of all types (e.g. AEs) in addition to treatment costs [1]. However, parameterization of the cost of AEs in economic models is often difficult due to limited data and inconsistent or incomplete methodological approaches [5-7]. This real-world study evaluated the incremental healthcare costs associated with AEs among patients diagnosed with 13 cancer types. Based on previous work by Wong et al., this study builds and expands our understanding of cancer-related costs by evaluating a larger number of AEs (54 vs 36), using an updated design schema (ICD-10 vs ICD-9), and a larger number of cancer types (13 vs 8).[5]. This study also flexibly (non-linearly) controls for the influence of co-occurring AEs on healthcare costs in addition to bias control requirements of exact matching on tumor type and propensity matching on patient/clinical characteristics, mitigating potential bias in a more systematic, robust manner than Wong et al.

The results of this study demonstrated that AE-related healthcare costs were considerable for both common and rare cancer types. Although there were commonalities in higher-cost AEs across common cancer types, the actual cost estimates can have a wide range depending on the cancer type. For example, generalized edema is noted as a costly AE in common cancers, with a range from $10,855 in breast cancer to $22,591 in colorectal cancer. Greater variation of AE costs was seen among rare cancer types. For example, the incremental cost per treatment episode of pancytopenia ranged from $31,090 in follicular lymphoma to -$5,704 in liver cancer. These results could be due in part to the inherently smaller patient numbers available for these cancers, making outlier values more influential on the reported means. Larger sample sizes will be needed to better stratify high- and low-cost AEs for rare cancer types. Finally, estimates from this study that are negative should not be interpreted as evidence that the AE in question is cost-saving, but rather as a reflection of the large, estimated

error due to the relatively small number of matched pairs. However, negative sample estimates are more likely to occur when the true population mean is closer to zero based on statistical intuition.

Severe AEs tend to cost more than less severe AEs due to the associated hospital stay inherent in the definition of a severe AE. This holds true in this analysis where the mean cost of any AE is $6,835 vs $9,518 for a severe AE. However, the costliest AE of any severity was $29,329 for allergic reaction and was considerably higher than the highest cost of severe AEs (GI bleeding, $19,525). This difference is in part due to the lack of estimate for severe allergic reaction, so no direct comparison is possible in this analysis. Additionally, severe AE estimates were generated from a much smaller set of treatment episodes that that for AEs of any severity, leading to greater variability of estimates.

There are several limitations in this study. Usage of standard propensity score matching assumes the validity of these results is conditional on the assumption that, after matching, there remain no factors that correlate to both the outcome (healthcare costs) and the treatment variable (the occurrence of the AE of interest). If this assumption is violated, that produces a source of bias in these results. Another limitation is that claims data contain limited information on the underlying cause of a diagnosis; therefore, AEs could not be directly linked to a treatment or underlying disease. Additionally, claims data do not provide information on the severity of disease (cancer stage) or course of disease. Finally, there was a limited sample of severe AEs and a limited number of many of the specific tumor types selected for analysis; as a result, there is greater uncertainty in our estimates of the cost of AEs in these populations.

~~5.~~ Conclusions

This study applied the design of Wong et al. to an updated patient data set with more tumor types and AEs and improved upon its methodology by applying the more up-to-date and more

accurate ICD10 diagnosis schema. By providing detailed estimates of the cost of common, cancer-related AEs, this study improves the ability of health economists to accurately characterize incremental cost differences between different regimens for the treatment of cancer.

Acknowledgments

The authors are grateful to Justin Nedzesky for early contributions he made to the design of this study. We are also grateful to Will Wong for his feedback on the study protocol and on early versions of this manuscript. Medical writing support provided by Lidija Garan, PhD, of Nucleus Global, an Inizio company.

Author contributions

- Conceptualization: Jesse Sussell
- Data curation: Achal Patel, Robert Schuldt
- Formal analysis: Achal Patel, Robert Schuldt
- Funding acquisition: NA
- Methodology: Achal Patel, Robert Schuldt, Jesse Sussell
- Project administration: Achal Patel, Robert Schuldt, Jesse Sussell
- Software: Achal Patel, Robert Schuldt
- Supervision: Jesse Sussell
- Validation: Achal Patel, Robert Schuldt

- Visualization: Achal Patel, Robert Schuldt, Jesse Sussell
- Writing – review & editing: Achal Patel, Robert Schuldt, Jesse Sussell

References

1. Garrison LP Jr, Neumann PJ, Willke RJ, Basu A, Danzon PM, Doshi JA, et al. A health economics approach to US value assessment frameworks-summary and recommendations of the ISPOR Special Task Force Report [7]. Value Health. 2018;21(2):161-5.

2. Arondekar B, Curkendall S, Monberg M, Mirakhur B, Oglesby AK, Lenhart GM, Meyer N. Economic burden associated with adverse events in patients with metastatic melanoma. J Manag Care Spec Pharm. 2015;21(2):158-64.

3. Copley-Merriman C, Stevinson K, Liu FX, Wang J, Mauskopf J, Zimovetz EA, Chmielowski B. Direct costs associated with adverse events of systemic therapies for advanced melanoma: systematic literature review. Medicine (Baltimore). 2018;97(31):e11736.

4. McGregor B, Geynisman DM, Burotto M, Porta C, Suarez C, Bourlon MT, et al. Grade 3/4 adverse event costs of immuno-oncology combination therapies for previously untreated advanced renal cell carcinoma. Oncologist. 2023;28(1):72-9.

5. Wong W, Yim YM, Kim A, Cloutier M, Gauthier-Loiselle M, Gagnon-Sanschagrin P, Guerin A. Assessment of costs associated with adverse events in patients with cancer. PLoS One. 2018;13(4):e0196007.

6. Abu SF, Shafie AA, Chandriah H. cost estimations of managing adverse drug reactions in hospitalized patients: a systematic review of study methods and their influences. Pharmacoepidemiology. 2023;2(2):120-39.

7. Durand M, Castelli C, Roux-Marson C, Kinowski JM, Leguelinel-Blache G. Evaluating the costs of adverse drug events in hospitalized patients: a systematic review. Health Econ Rev. 2024;14(1):11.

8. Shepard CW, Ortega-Sanchez IR, Scott RD, 2nd, Rosenstein NE, Team AB. Cost-effectiveness of conjugate meningococcal vaccination strategies in the United States. Pediatrics. 2005;115(5):1220-32.

9. Jewell EL, Kulasingam S, Myers ER, Alvarez Secord A, Havrilesky LJ. Primary surgery versus chemoradiation in the treatment of IB2 cervical carcinoma: a cost effectiveness analysis. Gynecol Oncol. 2007;107(3):532-40.

10. Richard P, Walker R, Alexandre P. The burden of out of pocket costs and medical debt faced by households with chronic health conditions in the United States. PLoS One. 2018;13(6):e0199598.

11. Borghaei H, Yim YM, Guerin A, Pivneva I, Shi S, Gandhi M, Ionescu-Ittu R. Severe adverse events impact overall survival and costs in elderly patients with advanced non-small cell lung cancer on second-line therapy. Lung Cancer. 2018;119:112-9.

12. Yim YM, Gandhi M, Guerin A, Ionescu-Ittu R, Pivneva I, Shi S, Wu E. Impact of severe adverse events during second-line therapy on healthcare costs in patients with advanced non-small cell lung cancer (aNSCLC). Ann Oncol. 2016;27:vi424.

Supporting information

S1 Fig. Correlation matrix for AEs of any severity in breast cancer cohort. AE, adverse event; CNS, central nervous system; GI, gastrointestinal. [a]Arrhythmia, i.e. arrhythmia/tachycardia/atrial fibrillation. [b]Pain includes myalgia, back/musculoskeletal/laryngeal pain, pain in extremities, proctalgia, pain in limb, abdominal pain, and neuritic pain. [c]Psychiatric disorders include depression, anxiety, confusion, agitation, euphoria, disorientation, emotional ability, hallucinations, mood alteration, nervousness, agitation, and irritability. [d]Thromboembolic events include pulmonary embolism, thrombotic/embolic stroke, and venous/arterial thrombolism/embolism.

S2 Fig. Correlation matrix for severe AEs[a] in breast cancer cohort. AE, adverse event. [a]Only severe AEs (AEs grade 3-4) with available data were evaluated. [b]Arrhythmia, i.e. arrhythmia/tachycardia/atrial fibrillation. [c]Pain includes myalgia, back/musculoskeletal/laryngeal pain, pain in extremities, proctalgia, pain in limb, abdominal pain, and neuritic pain. [d]Thromboembolic events include pulmonary embolism, thrombotic/embolic stroke, and venous/arterial thrombolism/embolism.

Figure 1

Click here to access/download;Figure;Fig1.tif

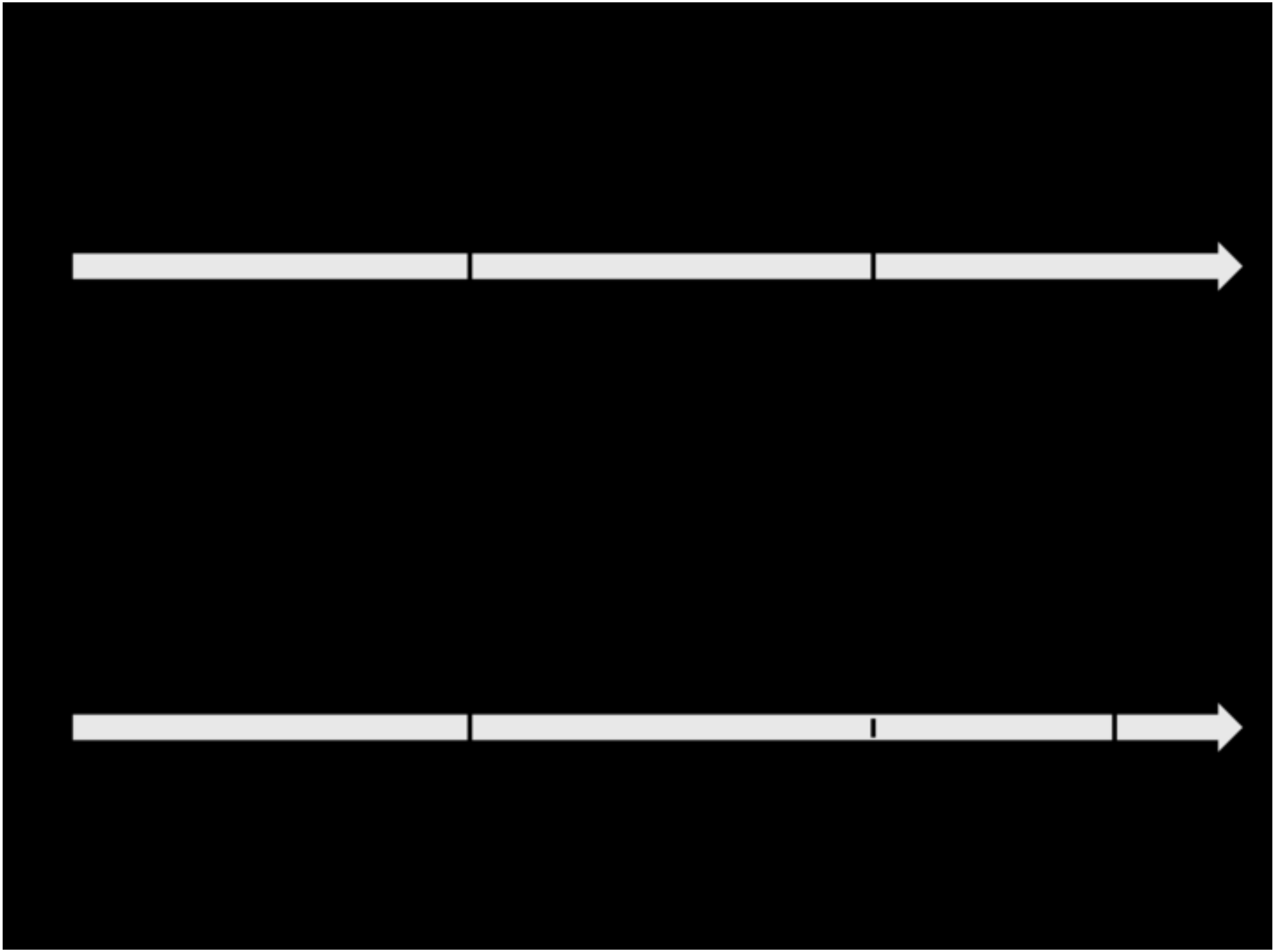

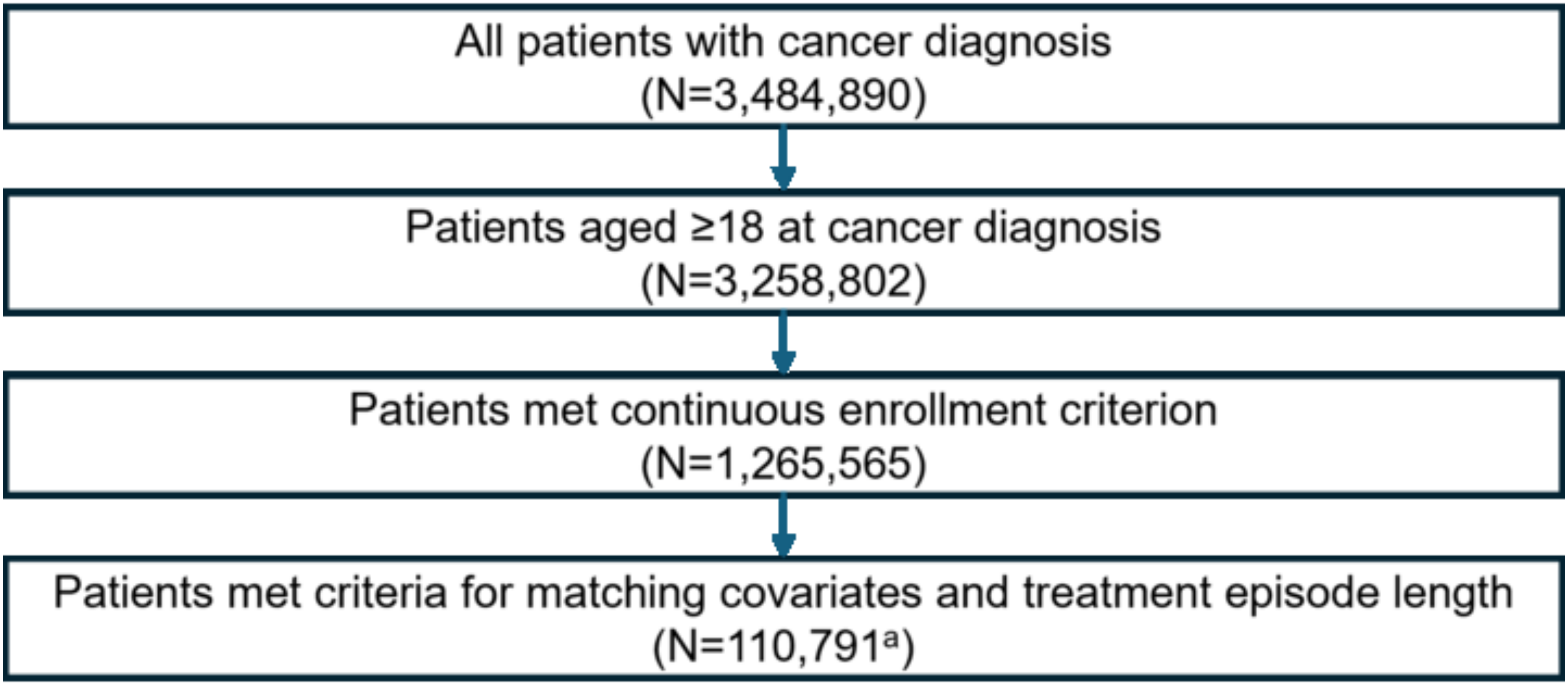
All patients with cancer diagnosis
(N=3,484,890)
Patients aged ≥18 at cancer diagnosis
(N=3,258,802)
Patients met continuous enrollment criterion
(N=1,265,565)
Patients met criteria for matching covariates and treatment episode length
(N=110,791ᵃ)

Figure 3

Click here to access/download;Figure;Fig3.tif

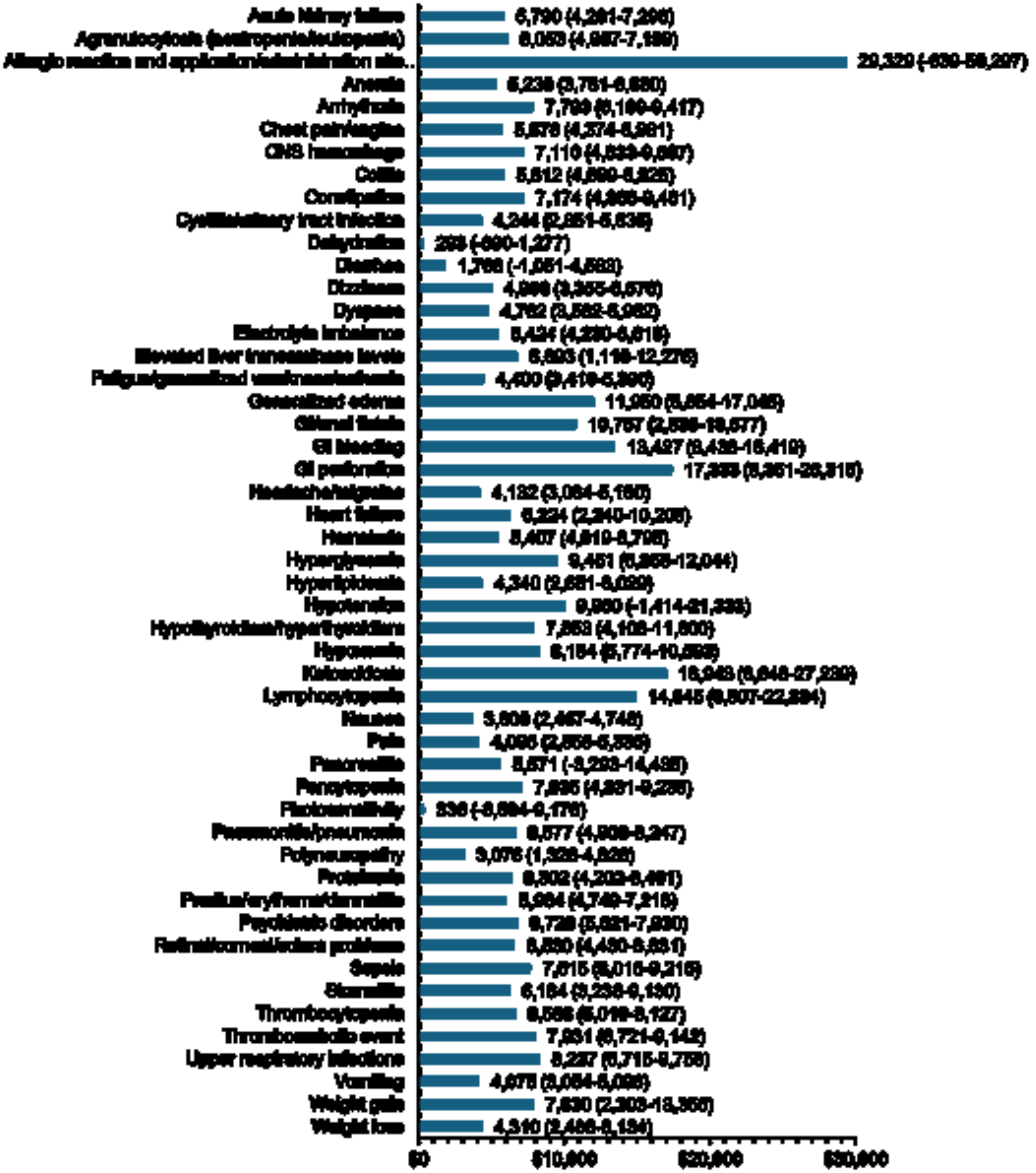

Figure 4

Click here to access/download;Figure;Fig4.tif

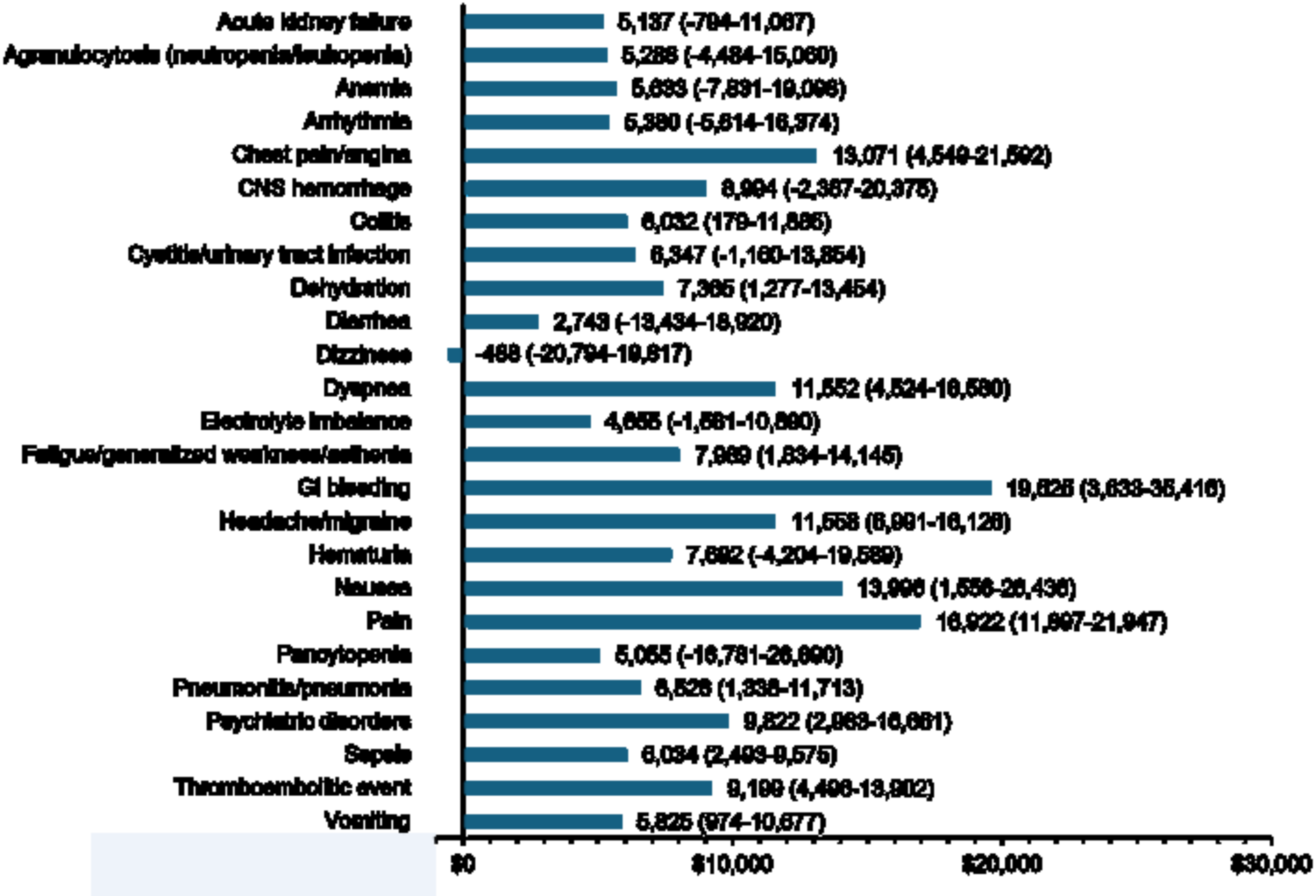

Supporting Information S1 Figure

Click here to access/download
Supporting Information
S1Fig.pdf

Supporting Information S2 Figure

Click here to access/download
Supporting Information
S2Fig.pdf
