# Supplementary material for: New estimates of the costs of adverse events in patients with cancer
Source: PLoS One. 2025 Sep 29;20(9):e0332703. doi: 10.1371/journal.pone.0332703 (PMC12478943; doi:10.1371/journal.pone.0332703)
Supplement: Attachment — Submitted filename: PLoS_One_ResponseToReviewers_03July2025.docx [file pone.0332703.s006.docx]

Reviewer #1: Thank you for the opportunity to re-review the manuscript. I believe most of my comments have been addressed. Two issues remain:

1. Diagnostic claims are claims associated with determining the diagnosis and include the initial imaging, biopsy, pathology tests and associated codes. Were these included in determine patient selection?

**Response:**

Thank you for this question. To clarify, patient selection was determined through the ICD10 diagnosis classification associated with the medical and pharmacy claims themselves. So for example, the patients in our lung cancer cohort had pharmacy/medical claims with the C34.x ICD10 codes listed in the diagnosis position.

It is true that many of these patients had diagnostic procedures associated with their conditions, and that these procedures were used to determine the actual diagnosis of lung cancer. For example, a patient with lung cancer may have had an MRI during the course of diagnosis. But a patient with COPD might also have an MRI. If this had been an analysis of Electronic Medical Record (EMR) data, this information could be utilized to inform patient selection / classification. But the nature of claims data is that at the time the MRI (or biopsy, or panel blood test) occurs, the actual diagnosis is not yet known: Someone who has a chest MRI may have lung cancer, or COPD, or any of several other conditions.

To summarize, we did not use the presence of specific diagnostic tests to determine patient cohort classification, because in claims data (at least at the beginning of the treatment journey) the information associated with those claims does not typically convey information about the patient's actual diagnosis.

2. There is a summary of a paper by Lal et al in the discussion, which is not summarized appropriately. The study population is not just Medicare Advantage; it also includes commercial patients.

**Response:**

Thank you for bringing this error to our attention. We have revised the manuscript text as follows: *A second study examined the costs of adverse events in patients with hepatocellular carcinoma, the most common type of liver cancer, in a pooled Medicare Advantage population. Their design differs from ours in that our study considers all types of liver cancer, including but not limited to HCC, thus precluding direct comparison of estimates.*
